# Greater perceived stress and lower self-regulation associated with substance use behaviors in a national sample of United States emergency medical service clinicians

**DOI:** 10.1016/j.drugalcdep.2026.113360

**Published:** 2026-09-10

**Authors:** Enzo G. Plaitano, Jonathan R. Powell, Christopher B. Gage, Ashish R. Panchal, Madelyn R. Frumkin, Lisa A. Marsch, Christine M. Gunn

**Affiliations:** a Center for Technology and Behavioral Health, Geisel School of Medicine, Dartmouth College, Lebanon, NH, USA; b The Dartmouth Institute for Health Policy and Clinical Practice, Geisel School of Medicine, Dartmouth College, Hanover, NH, USA; c Clinical and Research Services, ImageTrend Inc., Eagan, MN, USA; d Research Team, National Registry of Emergency Medical Technicians, Columbus, OH, USA; e Department of Emergency Medicine, Wexner Medical Center, The Ohio State University, Columbus, OH, USA

**Keywords:** Emergency medical service, Perceived stress, Self-regulation, Alcohol binge drinking, Cannabis, Nicotine

## Abstract

**Background:**

Emergency medical service (EMS) clinicians are first responders who can experience work-related stressors. Substances can be used as a coping strategy to manage stress. The ability to self-regulate motivation, cognition, and emotions may protect against substance use. Our objective was to examine the associations among stress, self-regulation, and substance use in EMS clinicians to identify potential psychological targets for substance use prevention.

**Methods:**

We invited a random sample of 19,000 full-time United States EMS clinicians from the largest national EMS database, of whom 1510 completed the survey. The online survey distributed by email included validated scales for perceived stress and self-regulation, as well as items assessing the number of days with binge drinking, cannabis use, and nicotine use in the prior month. We examined substance use outcomes using multivariable zero-inflated negative binomial models weighted to reflect population sociodemographic distributions.

**Results:**

Each 1-point increase in perceived stress was associated with 21% more binge drinking days (aIRR=1.21 [1.05, 1.39]), 79% more cannabis use days (aIRR=1.79 [1.22, 2.61]), and 5% more nicotine use days (aIRR=1.05 [1.01, 1.10]). In contrast, each 1-point increase in self-regulation was associated with 97% lower odds of using cannabis at all (aOR=0.03 [0.01, 0.57]) and 33% fewer binge drinking days (aIRR=0.67 [0.51, 0.88]).

**Conclusions:**

In this national cross-sectional study of full-time United States EMS clinicians, we identified stress and self-regulation as factors significantly associated with substance use. Future studies should determine whether workplace and individualized interventions targeting perceived stress and self-regulation help reduce substance use among EMS clinicians.

## 1. Introduction

Emergency medical service (EMS) clinicians are first responders who deliver life-saving care in the prehospital setting (Ehlers et al., 2023; Panchal et al., 2022). Their essential job-related tasks include evaluating patients, identifying and treating life-threatening conditions, administering medications, performing procedural interventions, and transporting patients to the hospital (Ehlers et al., 2023; Panchal et al., 2022). EMS clinicians perform these essential job tasks while only receiving an average wage of $17 per hour (U.S. Bureau of Labor Statistics, 2025). In the process of delivering this high-intensity prehospital care, EMS clinicians are frequently placed in isolated and unpredictable situations (e.g., extreme weather, unsafe buildings, remote forests) often with limited access to life-saving equipment, sufficient personnel, and medical oversight (Alghamdi, 2022; Bonumwezi et al., 2022; Huang et al., 2022; Mountfort and Wilson, 2022).

EMS clinicians report higher rates of substance use during their EMS careers compared to before entering the field (Basting et al., 2023). Stress is related to the use of alcohol (Keyes et al., 2011; Sinha, 2022), cigarettes (McKee et al., 2003; Slopen et al., 2013), and cannabis (Glodosky and Cuttler, 2020) among other populations of adults. Repeated exposure to difficult situations can increase stress, which could lead some first responders to use substances as a maladaptive coping strategy (Gill and Lauren, 2025; Huang et al., 2022). Specifically, the Self-Medication Hypothesis suggests that individuals may use substances to alleviate distress and tension (Khantzian, 1985). While this can provide short-term relief, reliance on this strategy over time can contribute to substance use disorders and dependence, regardless of stressors (Khantzian, 1985).

In one study, 33% of the 3000 first responders surveyed reported using substances to manage their emotional distress (Witkowski et al., 2023). The most frequently endorsed substances among first responders are alcohol, cannabis, and nicotine, with substance co-use commonly reported (Gabay et al., 2025). Specifically, firefighters who smoke cigarettes are five times more likely to endorse binge drinking (Jitnarin et al., 2015, 2013) and significantly more likely to endorse cannabis use (Testoff et al., 2025) compared to those who do not smoke. While known associations exist between stress and higher likelihood of substance use among convenience samples of first responders (Arble and Arnetz, 2017; Gabay et al., 2025; Witkowski et al., 2023), studies have primarily included composite measures of substance use (e.g., the nonspecific use of any substances), which limits the identification of stress as a potential risk factor across different substance use behaviors (Arble and Arnetz, 2017; Gabay et al., 2025; Witkowski et al., 2023).

In contrast to stress, studies in general adult populations have found that greater self-regulation is associated with less substance use (González-Roz et al., 2024; Hand et al., 2024; Roos et al., 2020). Self-regulation is frequently defined as the ability to control motivations, cognition, and emotions to avoid immediate gratification and better achieve long-term goals (Eisenberg et al., 2018). Across first responders and other adult populations with health-risk behaviors, lower self-regulation has been associated with greater substance use risks (Arble and Arnetz, 2017; Chopko et al., 2013; de Ridder and de Wit, 2008; Díaz-Tamayo et al., 2022; Roos and Witkiewitz, 2017). Prior studies have only examined composite measures of substance use or only one substance in isolation. No studies of first responders have examined self-regulation and the use of individual substances, despite these substances all having different health risks, use patterns, and treatment options (Lee et al., 2020; Sokolovsky et al., 2020; Vergara et al., 2018).

Among all first responder groups, including career police officers and professional firefighters, EMS clinicians might be among those at the highest risk for experiencing mental health and substance use (Gill and Lauren, 2025; Huang et al., 2022). Therefore, in line with the Self-Medication Hypothesis (Khantzian, 1985), this cross-sectional study examined the associations of perceived stress and self-regulation with the frequency of prior-month binge drinking, cannabis use, and nicotine use in a national random sample of United States EMS clinicians. We hypothesized that greater perceived stress would be associated with more frequent substance use, whereas greater self-regulation would be associated with less frequent use. Since the most commonly endorsed substances among first responders include alcohol, cannabis, and nicotine, we specifically examined associations across these three substances (Gabay et al., 2025). These findings can help determine whether future interventions aimed at decreasing stress or increasing self-regulation might help reduce substance use behaviors among first responders.

## 2. Methods

### 2.1. Participants

Inclusion was limited to United States EMS clinicians aged ≥ 18 years who worked full time responding to 9–1–1 emergencies. We included EMS clinicians across the United States who provided independent patient care and held Emergency Medical Technician (EMT), Advanced EMT, or paramedic certifications. We excluded emergency medical responders since this certification level has a very limited scope of practice and only represents 2–3% of the national EMS database (Cash et al., 2021). We limited our inclusion criteria to EMS clinicians working in emergency care settings (e.g., immediate response to injuries and illness), because these settings involve greater exposure to occupational stressors (Cannuscio et al., 2016). We excluded individuals who only worked in community settings (e.g., vaccine clinics or physician offices).

### 2.2. Procedures

#### 2.2.1. Study approval and ethics

This cross-sectional study was conducted in collaboration with the National Registry of EMTs. All participants provided informed consent. No compensation was provided. This study was approved by the American Institutes of Research Institutional Review Board (#EX00678) and Dartmouth College Committee for Protection of Human Subjects (#00032966).

#### 2.2.2. Participant recruitment

The National Registry maintains the largest database of EMS clinicians in the United States, containing records for up to 95% of certified clinicians (Cash et al., 2021). We invited a simple random sample of 19, 000 EMS clinicians from the national EMS database who met the above eligibility criteria to complete an anonymous online survey distributed by email between April and June 2024. The sampling frame of 19,000 individuals was large due to low expected response rates in online surveys of EMS clinicians (5–8%) (Cash et al., 2019; Gregory et al., 2022; Melgoza et al., 2025; Panchal et al., 2022; Powell et al., 2025) and was designed to achieve our desired sample of 1205 based on statistical power calculations. The National Registry sent reminders two and four weeks after the initial email using the modified Dillman method (Hoddinott and Bass, 1986). The overall survey completion rate was 7.9% (n = 1510).

### 2.3. Measures

#### 2.3.1. Demographics

Sociodemographic variables were collected for covariate adjustments and included age, sex, race, ethnicity, education, and EMS certification (Rivard et al., 2021). A combined minority-status variable was created since individual racial and ethnic subgroups were small. We selected these covariates for adjustment since younger age, male sex, minority status, lower education, and occupational status are associated with different substance use risks across other populations (Ellis et al., 2024; Li et al., 2023). The National Registry provided de-identified demographic and occupational characteristics from their database for all participants (Rivard et al., 2021). EMS clinicians update their data every two years during their recertification cycle.

#### 2.3.2. Perceived stress

The Perceived Stress Scale is a 10-item validated measure of thoughts and feelings related to stress over the prior month (Cohen et al., 1983). Participants rated how often they experienced each item on a 5-point Likert scale from (0) “Never” to (4) “Very often.” The item scores were averaged to create a mean continuous score (range=0–4), where higher scores indicated greater stress (Cherry and Wilcox, 2020; Sischka et al., 2024). Internal consistency was excellent for perceived stress (α=0.91) in our sample.

#### 2.3.3. Self-regulation

A 12-item self-regulation scale measured the ability to control motivations, cognition, and emotions to avoid seeking immediate gratification and better achieve long-term goals during the prior month (Scherer et al., 2022). The scale assessed the ability to manage emotional experiences, direct attention and awareness, set and pursue goals, and regulate risky behaviors on a 5-point Likert scale from (1) “Not at all like you” to (5) “Extremely like you.” All items were scored as described in the measure development paper (Scherer et al., 2022). Item scores were then averaged to create an overall continuous mean score (1–5) for each participant, where higher scores indicated greater self-regulation. Internal consistency was good for self-regulation (α=0.85) in our sample.

#### 2.3.4. Substance use frequency

Our primary outcome of interest in the models was substance use frequency. Three separate items from the National Survey on Drug Use and Health were used to measure the number of days in the prior month that participants engaged in (1) alcohol binge drinking (defined in the survey as 4 + standardized drinks in one sitting for women and 5 + in one sitting for men), (2) cannabis/marijuana use, and (3) nicotine use (Substance Abuse and Mental Health Services Administration [SAMHSA], 2024). For example, the survey provided a definition of a standardized alcohol drink and then asked participants, “During the past month, on how many days did you have [branched: men = ‘5’; women = ‘4’] or more drinks on the same occasion? By ‘occasion,’ we mean at the same time or within a couple of hours of each other.” For each separate substance use item, participants entered the number of days that they used that substance as a count ranging from 0 (none) to 30 days (every day) within the prior month.

#### 2.3.5. Substance use motives

To better understand the motivation for substance use in EMS clinicians, we descriptively examined two substance use items from the Brief COPE. The Brief COPE is a validated measure of coping strategies used in response to stressful events, and was slightly adapted to ask how often participants engaged in substance use to cope with “stressful EMS work-related events” in the prior month (Carver, 1997). Participants rated how often they used alcohol or other drugs to feel better or to help them get through it on a scale from (1) “I haven’t been doing this at all” to (4) “I’ve been doing this a lot” (Carver, 1997). We first descriptively examined the extent to which EMS clinicians endorsed substance use to cope with work-related events (continuous score) and then dichotomized the items into never (score: 1) vs. at least some (scores: 2–4) to report on the proportion of participants who used substances to help manage their work stress.

### 2.4. Statistical analyses

#### 2.4.1. Software and missing data

Statistical analyses were conducted using Stata v.18.0 (StataCorp LLC., College Station, TX). To address missingness in covariates merged from the national EMS database, we used a missing-category (missing-indicator) approach and created a “missing/unknown” level to retain records with incomplete covariate data for participants. This approach helped preserve the sample size and minimize potential selection bias from more restrictive complete-case analysis.

#### 2.4.2. Survey weighting

In recent years, online survey response rates have been declining substantially, especially among healthcare professionals (Timmins et al., 2023). It is common to have response rates of 10–15% among healthcare professionals (Sammut et al., 2021), and 5–8% in EMS clinicians specifically (Cash et al., 2019; Gregory et al., 2022; Melgoza et al., 2025; Panchal et al., 2022; Powell et al., 2025). Therefore, these surveys have high risk for nonresponse bias (Lynn, 1996). Survey weighting to help correct for nonresponse bias is a common methodology to reproduce the known sociodemographic distributions from the random sampling frame and adjust for the under- or over-representation of certain respondents (Jensen et al., 2022). Therefore, we applied survey weighting to help account for nonresponse bias and increase the generalizability of our results to the broader workforce of United States EMS clinicians (Jensen et al., 2022). We calculated weights by comparing distributions of age, sex, minority status, education, and EMS certification among the 19,000 randomly sampled clinicians with those among the 1510 respondents. Composite base weights were assigned to individuals to account for numerous variables in the dataset. Our sampling frame was a simple random sample of the national EMS database, and thus intended to be representative of the larger EMS population.

#### 2.4.3. Primary models

We fit three survey-weighted multivariable zero-inflated negative binomial (ZINB) regression models. These included one for each substance-use outcome (e.g., alcohol binge drinking, cannabis use, and nicotine use). ZINB models were selected because the count outcomes were non-negative, right-skewed (Prost et al., 2021), overdispersed (e. g., variance ≥ mean) (Ver Hoef and Boveng, 2007), and contained excess zeros (Yang et al., 2010). The substance use frequency data (see Section 2.3.4 Substance Use Frequency) were entered into the ZINB model as count outcomes. The zero-inflation component modeled the odds of belonging to the excess-zero group. We inverted estimates from this component so that adjusted odds ratios represented the odds of any use of a certain substance (≥ 1 day/month) versus no use (0 days/month). Results from this component are presented as adjusted odds ratios (aORs) with 95% confidence intervals (CI) per 1-unit increase in the exposure. The negative-binomial count component estimated relative differences in the expected number of substance-use days after accounting for the excess-zero process (Ver Hoef and Boveng, 2007). Results from the count component are presented as adjusted incidence rate ratios (aIRRs) with 95% CIs and interpreted as the relative change in the expected number of days with substance use per 1-unit increase in the exposure.

#### 2.4.4. Adjusted covariates

All ZINB models adjusted for age, sex, minority status, education, and level of EMS certification because prior research identified these characteristics as potential confounders (Ellis et al., 2024; Li et al., 2023). Prior studies suggest that the use of one substance can confound associations with another substance-use outcome (Schweinsburg et al., 2011). Therefore, given high rates of alcohol, cannabis, and nicotine co-use in first responders (Gabay et al., 2025), we controlled for the use of other substances within each model. For example, when alcohol binge drinking was our primary outcome of interest, we also adjusted for use of cannabis and nicotine. Thus, the models estimated associations with each substance use outcome after accounting for use of other substances (Schweinsburg et al., 2011). Multicollinearity among exposures was assessed in the three substance use models by examining variance inflation factors (VIF). VIF scores ranged from 1.14 to 1.16 for all three models, indicating no problematic multicollinearity (Kim, 2019).

## 3. Results

### 3.1. Descriptive characteristics

The mean age of participants was 42.6 years (SD = 11.4, range 18–79 years) (Table 1). Most participants were male (75.4%), White (91.0%), and non-Hispanic (92.7%). In total, 11.3% were classified as members of a racial or ethnic minority group. Most participants were paramedics (66.0%) and 53.7% held an associate degree or higher. Mean perceived stress scores over the prior month were moderate (Mean=1.8, SD=0.8, range=1.0–4.0) (Cohen et al., 1983). Mean self-regulation over the prior month was moderate (Mean=3.2, SD=0.4, range=1.5–4.2) (Scherer et al., 2022).

### 3.2. Substance use frequency

In the prior month, 36.5% of participants reported alcohol binge drinking, 8.3% reported cannabis use, and 36.4% reported nicotine use (Table 2 and Fig. 1). Overall, 56.8% of participants reported at least one of the three substance-use outcomes. Regarding co-use, 7.2% endorsed binge drinking and cannabis, 28.7% endorsed binge drinking and nicotine, and 6.2% endorsed cannabis and nicotine. Among participants reporting each substance-use outcome, the mean number of use days was 5.4 for binge drinking, 13.5 for cannabis, and 24.2 for nicotine.

### 3.3. Substance use motives

When participants were asked if they used substances to help cope with stressful work events, 33.4% of participants reported using alcohol or other drugs to make themselves feel better, while 29.7% reported using alcohol or other drugs to help get through it. Among participants who endorsed each motive, the mean frequency was a “little-to-medium” amount at 2.61 (SD = 0.80) for using substances to feel better and 2.61 (SD = 0.78) for using substances to get through the event (Carver, 1997). In the full sample, the corresponding frequencies were classified as “little-to-no” amounts at 1.53 (SD = 0.88) and 1.47 (SD = 0.84), respectively (Carver, 1997).

### 3.4. Associations with alcohol binge drinking

Among the entire sample, neither perceived stress (aOR=1.25 [0.92, 1.69]) nor self-regulation (aOR=0.80 [0.44, 1.46]) was associated with the odds of reporting any prior-month binge drinking (Table 3). In the count component for prior-month binge drinking, each 1-point increase in perceived stress was associated with a 21% higher expected number of prior-month binge drinking days (aIRR=1.21 [1.05, 1.39]), while each 1-point increase in self-regulation was associated with a 33% lower expected number of binge-drinking days (aIRR=0.67 [0.51, 0.88]).

### 3.5. Associations with cannabis use

Among the entire sample, perceived stress was not associated with the odds of reporting any prior-month cannabis use (aOR=0.76 [0.31, 1.85]) (Table 3). However, each 1-point increase in self-regulation was associated with 97% lower odds of reporting any prior-month cannabis use (aOR=0.03 [0.01, 0.57]). In the count component for prior-month cannabis use, each 1-point increase in perceived stress was associated with a 79% higher expected number of prior-month cannabis use days (aIRR=1.79 [1.22, 2.61]), while self-regulation (aIRR=1.79 [0.87, 3.67]) was not significantly associated with the number of prior-month cannabis use days.

### 3.6. Associations with nicotine use

Among the entire sample, neither perceived stress (aOR=0.93 [0.76, 1.14]) nor self-regulation (aOR=0.88 [0.61, 1.28]) was associated with reporting any prior-month nicotine use. In the count component for prior-month nicotine use, each 1-point increase in perceived stress was associated with a 5% higher expected number of nicotine use days (aIRR=1.05 [1.01, 1.10]) (Table 3). However, in the count component for prior-month nicotine use, self-regulation was not associated with the number of prior-month nicotine use days (aIRR=1.11 [0.99, 1.22]).

## 4. Discussion

### 4.1. Summary of results

Greater perceived stress and lower self-regulation were modestly associated with more binge drinking days. Greater perceived stress was also associated with more cannabis and nicotine use days, although the associations with nicotine use were small in magnitude. Greater self-regulation was associated with lower odds of any cannabis use. However, the cannabis estimates were imprecise and should be considered preliminary as they warrant replication in a larger sample. Overall, these findings suggest that future research should further examine associations of these psychological factors with cannabis use and alcohol binge drinking among EMS clinicians.

### 4.2. Demographic characteristics

We recruited a random sample of EMS clinicians through the National EMS Database. Overall, respondents were similar to the national EMS population, but differed on several characteristics. Compared with the national EMS population, respondents included higher proportions who identified as White or Hispanic/Latino, held a bachelor’s degree or higher, and were certified as paramedics (Cash et al., 2021). The proportion of female participants was similar in the respondent sample and national population (23.6% vs. 24.2%) (Cash et al., 2021). Some studies suggest that rates of substance use might be higher among individuals identifying as non-White race and those with less education (Ellis et al., 2024; Li et al., 2023). Therefore, it is possible that rates of substance use and the resulting associations may be underestimated in our current sample compared to the overall population of EMS clinicians. However, data from the Substance Abuse and Mental Health Services Administration suggest that rates of alcohol use may actually be higher among individuals who identify as White race when compared to all other racial/ethnic groups (Center for Behavioral Health Statistics and Quality, 2021). Therefore, we cannot confidently state that rates of substance use within our sample differ from the EMS population. Additionally, full-time paramedics often have higher socio-economic status than EMTs (Cash et al., 2022), which may have contributed to underestimation of prevalence and smaller associations (Ellis et al., 2024; Li et al., 2023). EMS clinicians also report high rates of housing insecurity, food insecurity, and health care or insurance costs (Basting et al., 2023). Therefore, future studies should examine potential associations between social determinants of health and substance use among large, national samples of EMS clinicians.

### 4.3. Results in context

We identified a high prevalence of alcohol binge drinking and nicotine use in this national sample of EMS clinicians, where almost one-third endorsed co-use of both substances. In comparison, these substance use rates were higher than those reported among United States adults which used methods and measures similar to those used in our survey (Fig. 1) (SAMHSA, 2024). The Self-Medication Hypothesis suggests that one potential reason why individuals might engage in substance use behaviors is as a maladaptive coping strategy to alleviate distress, tension, or withdrawal symptoms from substances (Khantzian, 1985). Given that EMS clinicians work in difficult environments (Bentley et al., 2013) and can experience greater occupational stress than workers in other occupations (Gill and Lauren, 2025), they might be using legal and accessible substances (e.g., alcohol and nicotine) to help manage their stress.

Despite higher rates of binge drinking and nicotine use, only 8.3% of respondents reported prior-month cannabis use, which is lower than the 16.3% prevalence in United States adults (Fig. 1) (SAMHSA, 2024). EMS clinicians may be more likely than other adults to abstain from cannabis use given greater stigma among healthcare professionals (Coughenour et al., 2021) and continued illegality or workplace restrictions in some states. While laws vary, first responders are often subject to routine job-related drug testing, which might also contribute to lower cannabis use (SAMHSA, 2024). In a large national study of working adults, individuals from workplaces that performed routine drug testing reported lower rates of cannabis use (Carpenter, 2007). However, stigmatized behaviors are also associated with underreporting (Binkowska et al., 2025). Some respondents may have underreported cannabis use because of social-desirability concerns (Binkowska et al., 2025). Additionally, some respondents may have chosen not to disclose potentially illicit behaviors (e.g., depending on state and workplace policies) in a survey associated with their EMS certification. Therefore, rates of substance use within this survey could be influenced by both lower prevalence and underreporting of use.

In adjusted analyses, increased perceived stress was not associated with higher odds of any prior-month use (versus none) among EMS clinicians. However, the count components indicated that greater perceived stress was associated with more alcohol binge drinking, cannabis use, and nicotine use days. Therefore, perceived stress may be significantly associated with more *days* of use among individuals who already use these substances. One possible explanation is that stress reinforces substance use craving (Ruisoto and Contador, 2019). Acute stress responses can disrupt the brain regions responsible for inhibitory control, which can contribute to substance use craving and increased use (Sinha, 2024). Together, our results suggest that intervention studies should determine whether stress-management interventions can reduce substance-use frequency.

Associations were stronger for cannabis use and binge drinking than for nicotine use. These associations can be contextualized through frequency of use. EMS clinicians reported more *episodic* binge drinking (5 days/month) and cannabis use (14 days/month). However, EMS clinicians reported *frequent* nicotine use (24 days/month), consistent with a more habitual pattern of nicotine use (Benowitz, 2010). Therefore, EMS clinicians may engage in episodic binge drinking or cannabis use in response to increased perceived stress. This is consistent with prior epidemiological studies, which found that up to 50% of people (Han et al., 2023) who use nicotine develop dependence, while only approximately 10% of individuals develop alcohol dependence and 7–8% develop cannabis dependence (Esser et al., 2014; Kritikos et al., 2025). In other populations, participants also reported that they engaged in more cannabis use (Cuttler et al., 2018) and binge drinking (Révész et al., 2022) when their stress or anxiety levels were high.

Lastly, in adjusted analyses, greater self-regulation was significantly associated with lower odds of using any cannabis and fewer days with binge drinking in the prior month. Given the limited number of individuals who used cannabis in this sample and the concerns for model instability (e.g., wide confidence intervals), our results should be considered preliminary until replicated in other samples. However, in other populations, individuals do report cannabis use more often than any other substance as a coping tool to deal with negative emotions when self-regulation was lower (Révész et al., 2022). This might explain why greater self-regulation was associated with lower odds of using any cannabis in the prior month among all respondents. Together, our results also suggest that future intervention studies should determine whether increasing self-regulation can reduce cannabis use and binge drinking among EMS clinicians.

### 4.4. Implications and directions for future research

Our findings, together with prior studies (Gabay et al., 2025; Huang et al., 2022), suggest that EMS clinicians might be at greater risk for substance use than the general public (SAMHSA, 2024). Therefore, EMS clinicians may benefit from interventions tailored to their unique work environment. For example, restrictions on workplace cigarette smoking can be effective for the general public (Centers for Disease Control and Prevention, 2014; Cummings, 2016; Meza et al., 2023), but restrictions (International Association of Fire Fighters, 2019) are often less effective for first responders (Phan et al., 2022). Targeting psychological factors, such as perceived stress and self-regulation, through tailored interventions might reduce substance use in this sample.

Workplace resilience programs have been shown to improve self-regulation (e.g., goal setting, awareness) and adaptive coping among first responders (Grill et al., 2019). Additionally, general workplace support strategies, including providing dedicated spaces to decompress while at work, are negatively associated with problematic substance use in first responders, possibly by reducing their perceived stress levels (Witkowski et al., 2023). Given that alcohol and nicotine can be used to promote social bonding among first responders (e.g., sharing cigarettes while at work), interventions tailored to these unique social-occupational risks among first responders may be critically important (Haddock et al., 2022; Jitnarin et al., 2021). Finally, access to licensed mental health clinicians can reduce frontline worker stress (Rosen et al., 2022). Numerous therapies, such as dialectical behavior therapy, cognitive behavioral therapy, and mindfulness-based cognitive therapy, can help improve self-regulation (Jadhakhan et al., 2022).

However, 33% of first responders reported that stigma prevents them from seeking mental health care (Haugen et al., 2017). First responders are concerned about confidentiality, potential career repercussions, scheduling appointments with rotating schedules, and a lack of available resources (Haugen et al., 2017). Given these barriers, digital health apps and web-based interventions can provide convenient, confidential, and immediate access to stress management and self-regulation interventions (Knaak et al., 2017). Digital health interventions have been deployed to reduce perceived stress (Linardon et al., 2024, 2019), improve self-regulation (Jadhakhan et al., 2022) and decrease substance use in other populations (Staiger et al., 2020). Although stress may be unavoidable, future studies should investigate the acceptability and feasibility of digital health interventions among EMS clinicians. EMS agencies and healthcare systems should consider testing digital tools for EMS clinicians to lower substance use risks.

## 5. Limitations

Despite the strengths of this study, we must consider limitations. First, while the Self-Medication Hypothesis is one explanation for substance use in EMS clinicians, other potential mechanisms include (1) genetic predisposition (e.g., Quantitative Genetic Theory) (Deak and Johnson, 2021), (2) social reinforcement, such as drinking together after difficult shifts (e.g., Social Learning Theory) (Smith, 2021), and (3) interacting genetic, psychological, and social factors (e.g., Biopsychosocial Model) (Feingold and Tzur Bitan, 2022). Therefore, future research should examine genetic and social factors among EMS clinicians. Similarly, future studies should adjust for potential psychological (e.g., post-traumatic stress disorder) and occupational (e.g., hours worked) confounders (Carey et al., 2011; Witkowski et al., 2023) in the analyses, as these factors have been associated with greater mental health and substance use risks among first responders.

Second, this cross-sectional design does not permit causal inference since all measures were anchored to the prior month. We could also not perform cross-sectional mediation analyses since those methods can produce biased estimates because the temporal ordering of the variables cannot be established (Pek and Hoyle, 2016). Given the temporal ambiguity from the cross-sectional survey design, it is also possible that more substance use contributes to higher perceived stress or lower self-regulation. There are likely reverse or bidirectional associations in which substance use can also impact stress and self-regulation (Juliano et al., 2025). Therefore, future studies should also use longitudinal designs to examine temporality and directionality.

Third, although our total sample size was large and our response rate was in line with other online studies of EMS clinicians (Cash et al., 2019; Gregory et al., 2022; Melgoza et al., 2025; Panchal et al., 2022; Powell et al., 2025), nonresponse bias is likely. Respondents were more likely to identify as White race, have higher education, and be certified as paramedics compared to the National EMS Database (Cash et al., 2021). These factors may be associated with higher socioeconomic status (Cash et al., 2022) and lower rates of substance use (Ellis et al., 2024; Li et al., 2023). Therefore, it is possible that this nonresponse bias contributed to underestimated substance use prevalence and smaller effect sizes in this particular sample of EMS clinicians. It is widely accepted that national, online surveys have nonresponse bias (Lynn, 1996), so we recruited a large, random sample and performed survey weighting, which may help reduce the potential for nonresponse bias and improve sample representativeness (Jensen et al., 2022).

Fourth, substance use is subject to recall and social desirability bias (Binkowska et al., 2025). Some participants may have underreported their substance use, and specifically cannabis use, which would underestimate both the prevalence and strength of our associations. While the National EMS database allowed us to recruit a national, random sample and perform robust survey weighting, we suggest that future studies determine whether recruiting EMS clinicians through independent sources (e.g., social media advertisements, online blog posts) results in significantly different prevalence rates of cannabis use compared to our current random sample. Additionally, the small number of participants reporting cannabis use may have contributed to the large observed confidence intervals and could indicate model instability. Therefore, we should interpret the effect sizes for cannabis use with caution until subsequent studies are conducted. We consider cannabis estimates to be preliminary until replicated in future studies.

Fifth, while we invited a random, nationally representative sampling frame of EMS clinicians, our survey respondents were predominantly White and non-Hispanic. The survey weights likely reduced some nonresponse bias but cannot entirely eliminate this threat to external validity (Jensen et al., 2022). Given that rates of substance use can be higher for individuals identifying as non-White race (Li et al., 2023), future studies should also consider oversampling individuals from racial and ethnic minority groups to increase generalizability to the entire EMS population.

## 6. Conclusions

This cross-sectional study identified psychological factors associated with substance use in a national sample of United States EMS clinicians. Greater perceived stress was significantly associated with more days of binge drinking, cannabis use, and nicotine use. In contrast, greater self-regulation was significantly associated with lower odds of endorsing any cannabis use at all and fewer days of binge drinking. Future longitudinal studies should examine temporal patterns, while future intervention studies should determine whether workplace and tailored interventions targeting both perceived stress and self-regulation reduce substance use among EMS clinicians.

## Figures and Tables

**Fig. 1.**
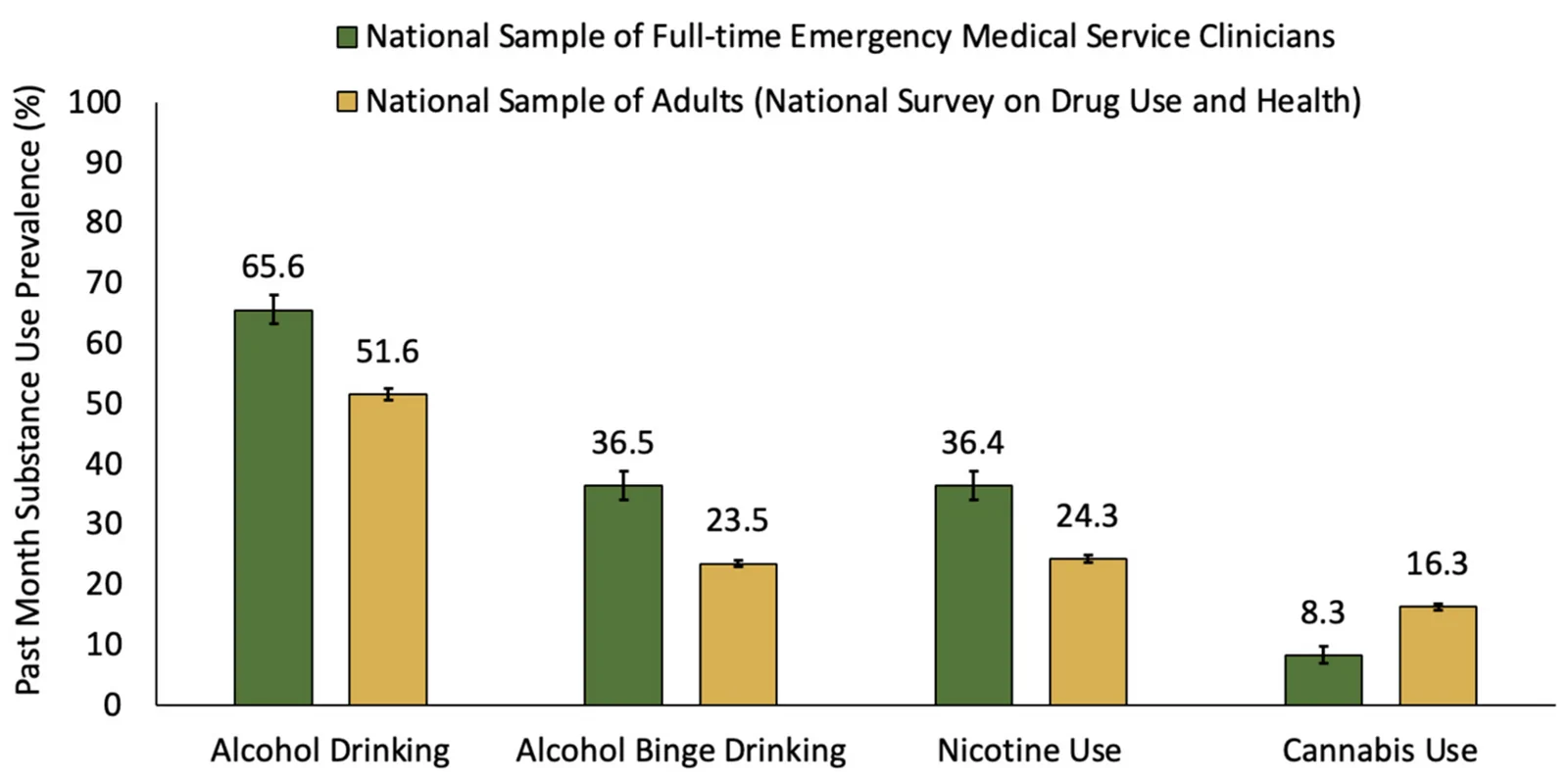
Prevalence of substance use in a national sample of United States emergency medical service clinicians compared to national data among United States adults.

**Table 1:** Participant descriptive characteristics.

Characteristic	
**Age in years,** *mean (SD)*	42.6 (11.4)
**Sex, n (%)**	
Female	357 (23.6)
Male	1138 (75.4)
Other/prefer not to say	15 (1.0)
**Race, n (%)**	
Indigenous American or Alaska Native	19 (1.3)
Asian	7 (0.5)
Black or African American	32 (2.1)
Native Hawaiian or Pacific Islander	7 (0.5)
White	1374 (91.0)
Prefer not to say	71 (4.7)
**Ethnicity, n (%)**	
Hispanic or Latino	110 (7.3)
Not Hispanic or Latino	1400 (92.7)
**Minority status** ^a^ **, n (%)**	
Minority	170 (11.3)
Not a minority	1340 (88.7)
**Education, n (%)**	
High school graduate/GED	181 (12.0)
Some college	502 (33.2)
Associate degree	344 (22.8)
Bachelor’s degree or higher	467 (30.9)
Unknown	16 (1.1)
**EMS certification, n (%)**	
EMT or Advanced EMT	514 (34.0)
Paramedic	996 (66.0)

Note: Categorical or dichotomous data are reported as n (%). Continuous data are reported as mean (SD);

a Minority status variable was defined as Non-White race and/or Hispanic or Latino ethnicity. EMT = Emergency Medical Technician; EMS = Emergency Medical Service; GED = General Educational certification.

**Table 2:** Mental health and substance use characteristics.

Characteristic	
**Perceived stress,** *mean (SD)*	1.8 (0.8)
**Self-regulation,** *mean (SD)*	3.2 (0.4)
**Any prior-month substance use,**^a^ **n (%)**	
Any binge drinking, cannabis use, or nicotine use	858 (56.8)
Alcohol binge drinking	551 (36.5)
Cannabis use	125 (8.3)
Nicotine use	550 (36.4)
**Days in prior month with substance use,**^b^ *mean (SD)*	
Alcohol binge drinking	5.4 (6.0)
Cannabis use	13.5 (11.0)
Nicotine use	24.2 (10.2)
**Any substance use to cope,**^a^ **n (%)**	
To feel better after work-related stress	504 (33.4)
To help cope with work-related stress	447 (29.7)
**Frequency of substance use motives,**^c^ *mean (SD)*	
To feel better after work-related stress	2.61 (0.80)
To help cope with work-related stress	2.61 (0.78)

Note: Categorical or dichotomous data are reported as n (%). Continuous data are reported as mean (SD).

a Individuals could endorse more than 1 response option for substance use, so the total number of participants and percentages in the categories can exceed 100%.

b Among participants reporting at least one day of use of the corresponding substance.

c Among participants endorsing the corresponding motive.

**Table 3:** Weighted associations of perceived stress and self-regulation with substance use.

Characteristic	Binge Drinking Prior Month		Cannabis Use Prior Month		Nicotine Use Prior Month	
	Any use vs. none entire sample	Expected number of use days, count	Any use vs. none entire sample	Expected number of use days, count	Any use vs. none entire sample	Expected number of use days, count

	aOR (95% CI)	aIRR (95% CI)	aOR (95% CI)	aIRR (95% CI)	aOR (95% CI)	aIRR (95% CI)
Perceived stress	1.25 (0.92, 1.69)	1.21 (1.05, 1.39)*	0.76 (0.31, 1.85)	1.79 (1.22, 2.61)*	0.93 (0.76, 1.14)	1.05 (1.01, 1.10)*
Self-regulation	0.80 (0.44, 1.46)	0.67 (0.51, 0.88)*	0.03 (0.01, 0.57)*	1.79 (0.87, 3.67)	0.88 (0.61, 1.28)	1.11 (0.99, 1.22)
Age, in years	0.96 (0.94, 0.98)*	1.01 (1.01, 1.02)*	0.95 (0.88, 1.02)	1.01 (0.98, 1.03)	0.97 (0.96, 0.98)*	1.00 (1.00, 1.01)
Male sex	1.43 (0.86, 2.35)	1.01 (0.79, 1.27)	1.41 (0.37, 5.57)	0.58 (0.33, 1.02)	1.37 (1.02, 1.85)*	0.98 (0.90, 1.06)
Minority status	0.79 (0.34, 1.84)	1.09 (0.79, 1.52)	1.52 (0.19, 12.87)	1.16 (0.57, 2.39)	1.05 (0.72, 1.54)	1.14 (1.01, 1.30)*
Education	0.85 (0.68, 1.07)	1.09 (0.99, 1.21)	1.28 (0.59, 2.78)	1.23 (0.86, 1.75)	0.69 (0.62, 0.79)*	0.94 (0.91, 0.97)*
Paramedic	1.39 (0.87, 2.24)	0.94 (0.76, 1.17)	1.23 (0.43, 3.57)	1.72 (1.12, 2.64)*	0.91 (0.70, 1.18)	0.96 (0.90, 1.02)
Binge drinking	-	-	6.16 (1.45, 34.18)*	0.80 (0.66, 0.98)*	1.49 (1.33, 1.67)*	1.00 (0.98, 1.02)
Cannabis use	Non-estimable^a^	0.92 (0.86, 0.98)*	-	-	1.14 (1.03, 1.23)*	1.00 (0.97, 1.02)
Nicotine use	1.07 (0.99, 1.15)	1.08 (1.04, 1.12)*	0.93 (0.72, 1.20)	1.10 (1.01, 1.21)*	-	-

Note. Survey weights were derived from the sampling frame (N = 19,000) and applied to the respondent sample (n = 1510). All models adjusted for the covariates shown. An asterisk indicates that the 95% CI does not include 1.00.

a Of note, cannabis use perfectly predicted binge drinking in the zero-inflation component of the binge-drinking model, resulting in a nonestimable coefficient.

## Data Availability

The raw data supporting the conclusions of this article are not yet publicly available as they are an essential component of a doctoral thesis that is still in progress. In the interim, the raw data supporting the conclusions of this article will be made available to individuals by the corresponding author upon reasonable request and will be public in the future.

## Glossary

(EMS): Emergency Medical Service
(EMT): Emergency Medical Technician
(ZINB): zero-inflated negative binomial regression
